# Triethyl­ammonium *O*-3β-cholest-5-en-3-yl (4-meth­oxy­phen­yl)dithio­phospho­nate

**DOI:** 10.1107/S1600536810029703

**Published:** 2010-07-31

**Authors:** Hendriette Van Der Walt, Alfred Muller, Werner E. van Zyl

**Affiliations:** a Department of Chemistry, University of Johannesburg (APK Campus), PO Box 524, Auckland Park, Johannesburg 2006, South Africa; b School of Chemistry, University of KwaZulu-Natal, Westville Campus, Private Bag X54001, Durban 4000, South Africa

## Abstract

In the crystal structure of the title compound, C_6_H_16_N^+^·C_34_H_52_O_2_PS_2_^−^ or [(CH_3_CH_2_)_3_NH]^+^·[C_34_H_52_O_2_PS_2_]^−^, the cation and anion are paired *via* weak, inter­molecular, bifurcated N—H⋯(S,S) hydrogen bonds. The cholesteryl units form an alternating (herringbone) motif as well as an infinitely stacked layered structure along the *b* axis. The P—S bond lengths [1.975 (2) and 1.981 (2) Å compared with *ca* 1.92 Å for a formal P=S double bond and with *ca* 2.01 Å for a P—S single bond] suggest delocalization of the negative charge between the P—S bonds. A distorted tetra­hedral geometry around the P atom is revealed by non-ideal O—P—C and S—P—S bond angles of 96.7 (2) and 115.52 (11)°, respectively.

## Related literature

For applications of dithio­phospho­nate derivatives, see: Beaton *et al.* (1991 ▶); Patnaik (1992 ▶); Roy (1990 ▶); Bromberg *et al.* (1993 ▶); Klaman (1984 ▶). For information on dithio­phospho­nate compounds, see: van Zyl *et al.* (1998 ▶, 2000 ▶, 2002 ▶); van Zyl *et al.* (2010 ▶). For P/S activation of steroids, see: Kvasnica *et al.* (2008 ▶). For related structures, see: Malenkovskaya *et al.* (2003 ▶); Cea-Olivares *et al.* (1999 ▶); Blaszczyk *et al.* (1996 ▶).
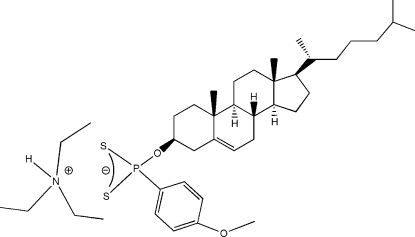


## Experimental

### 

#### Crystal data

C_6_H_16_N^+^·C_34_H_52_O_2_PS_2_^−^
*M* *_r_* = 690.04
Monoclinic, 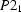

*a* = 7.6066 (15) Å
*b* = 8.2407 (16) Å
*c* = 33.083 (7) Å
β = 93.17 (3)°
*V* = 2070.6 (7) Å^3^
*Z* = 2
Mo *K*α radiation
μ = 0.20 mm^−1^
*T* = 293 K
0.46 × 0.08 × 0.08 mm

#### Data collection

Bruker SMART 1K CCD diffractometer
Absorption correction: multi-scan (*SADABS*; Bruker, 1998 ▶) *T*_min_ = 0.914, *T*_max_ = 0.984
14589 measured reflections
8425 independent reflections
2925 reflections with *I* > 2σ(*I*)
*R*_int_ = 0.106

#### Refinement

*R*[*F* ^2^ > 2σ(*F* ^2^)] = 0.066
*wR*(*F* ^2^) = 0.159
*S* = 0.93
8425 reflections
410 parameters
1 restraint
H atoms treated by a mixture of independent and constrained refinement
Δρ_max_ = 0.29 e Å^−3^
Δρ_min_ = −0.28 e Å^−3^
Absolute structure: Flack (1983 ▶), 2970 Friedel pairs
Flack parameter: 0.02 (12)

### 

Data collection: *SMART-NT* (Bruker, 1998 ▶); cell refinement: *SAINT-Plus* (Bruker, 1999 ▶); data reduction: *SAINT-Plus* and *XPREP* (Bruker, 1999 ▶); program(s) used to solve structure: *SIR2002* (Burla *et al.*, 2003 ▶); program(s) used to refine structure: *SHELXL97* (Sheldrick, 2008 ▶); molecular graphics: *DIAMOND* (Brandenburg & Brendt, 2001 ▶); software used to prepare material for publication: *WinGX* (Farrugia, 1999 ▶).

## Supplementary Material

Crystal structure: contains datablocks global, I. DOI: 10.1107/S1600536810029703/cv2748sup1.cif

Structure factors: contains datablocks I. DOI: 10.1107/S1600536810029703/cv2748Isup2.hkl

Additional supplementary materials:  crystallographic information; 3D view; checkCIF report

## Figures and Tables

**Table 1:** Hydrogen-bond geometry (Å, °)

*D*—H⋯*A*	*D*—H	H⋯*A*	*D*⋯*A*	*D*—H⋯*A*
N—H1⋯S1^i^	0.96 (7)	2.53 (7)	3.426 (7)	156 (5)
N—H1⋯S2^i^	0.96 (7)	2.78 (7)	3.437 (6)	126 (5)

Symmetry code: (i) 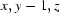.

**Fig. 1.**
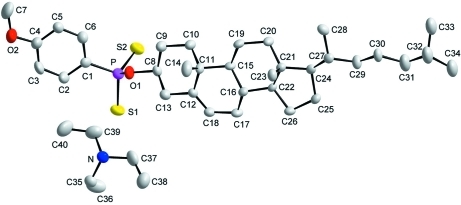
View of (I) showing the atomic numbering and 30% probability displacement ellipsoids. Hydrogen atoms omitted for clarity.

**Fig. 2.**
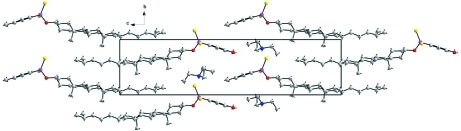
Packing diagram of (I) viewed along the a axis illustrating the herring-bone motif.

**Fig. 3.**
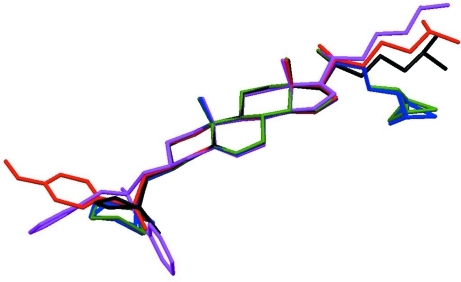
Superimposed (I) with the cholesterylphosphate structures available from the literature.

## supplementary crystallographic information

### Comment

The dithiophosphonato monoanion, [S_2_PR(OR')]^-^ may be described as a hybrid between the related dithiophosphato [S_2_P(OR)_2_]^-^ and dithiophosphinato [S_2_P*R*_2_]^-^ species. Of these, the dithiophosphonato version is of most interest for the following reasons: i) it can be considered rare in the chemical literature, particularly as a species that P/S activate natural products such as steroids (described in this study), and indeed for the majority of main- and transition metals simply non-existent, ii) from the reaction between a common precursor (usually Lawesson's Reagent), and any compound that contains a 1° or 2° alcohol functionality, a tremendous number of new and varied derivatives can be obtained in a facile manner, iii) the synthetic methodology allows for control in the design of the compound to perform reactions and yield new products in both organic and aqueous phases, and iv) solution and solid state ^31^P{^1^H} NMR spectroscopy is a valuable tool to obtain mechanistic and structural information on these compounds (van Zyl *et al.*,  2010). In terms of application, this class of compound has demonstrated use in a variety of technological areas such as oligonucleotide synthesis (Beaton *et al.*, 1991), agricultural insecticides (Patnaik, 1992) and -pesticides (Roy, 1990), derivatives of metal ore extraction reagents (Bromberg *et al.*, 1993) and antioxidant additives in the oil and petroleum industry (Klaman, 1984). In future, advances of these compounds as well as their metal complexes will be forthcoming in areas such as materials- and medicinal chemistry. General and convenient methods to dithiophosphonate salt derivatives have been reported (van Zyl *et al.*, 2000).

In the title compound, (I) (Fig. 1), all bond lengths and angles are normal and comparable with those observed in the related structures (Malenkovskaya *et al.*, 2003; Cea-Olivares *et al.*, 1999; Blaszczyk *et al.*, 1996). Aminium cations link cholesteryl moieties to form infinitely stacked layers along the *b* axis, supported by N—H···S interactions (see Fig. 2 and Table 1).

Only a few examples of the cholesteryl phosphate moiety exists (CSD show six hits with four usable results). Superimposing these (see Fig. 3) show large variations on the periphery of the molecules due to various packing arrangements found in each. Most notable of these interactions is the two different conformations adopted by the pentane tail of the cholesteryl moiety. The two configurations are differentiated by one group showing interactions to phosphate moieties of the neighbouring molecules.

### Experimental

A 25-ml Schlenk tube was charged with commercially available (Aldrich) Lawesson's Reagent [(4-C_6_H_4_OMe)(P(S)S)_2_] (6 mmol, 1 molar equivalent) and placed under vacuum for 30 minutes. The solid was then heated to approx. 70 °C and commercially available (Aldrich) cholesterol (12 mmol, 2 molar equivalents) was added in one portion together with 2 ml dry toluene. The temperature was maintained at 70–75 °C until dissolution of all solids were observed, and then stirred for a further 10–20 minutes. At this stage the dithiophosphonic acid had formed and no attempt was made to isolate it. The heat source was removed and the solution was cooled to room temperature. After 30 minutes it was cooled down further to 0 °C with the aid of an ice bath. The acid can be readily deprotonated by adding a few drops (12 mmol in theory, but a slight excess is not detrimental) of triethylamine with vigorous agitation of the solution which led to formation of a white colored precipitate. The material was dried and consolidated with small additions of cold diethyl ether, and filtered on a frit. The isolated air-dried salt can be stored under a nitrogen atmosphere. The salt was dissolved in dichloromethane and layered with hexanes in a stoppered test-tube, but crystal growth proved slow and the test-tube stopper was subsequently removed, allowing the solvents to slowly evaporate at room temperature which led to the growth of a sufficient number of single crystals suitable for X-ray diffraction analysis.

### Refinement

The aromatic, methine, methylene and methyl H atoms were placed in geometrically idealized positions (C—H = 0.97–0.98 Å) and constrained to ride on their parent atoms with *U*_iso_(H) = 1.2*U*_eq_(C) for the aromatic, methylene and methine H and *U*_iso_(H) = 1.5*U*_eq_(C) for the methyl H respectively. Torsion angles for the methyl H were refined from electron density. The aminium H was located in a Fourier difference map and refined isotropically.

### Crystal data

**Table:**

C_6_H_16_N^+^·C_34_H_52_O_2_PS_2_^−^	*F*(000) = 756
*M**_r_* = 690.04	*D*_x_ = 1.107 Mg m^−^^3^
Monoclinic, *P*2_1_	Mo *K*α radiation, λ = 0.71073 Å
Hall symbol: P 2yb	Cell parameters from 714 reflections
*a* = 7.6066 (15) Å	θ = 2.5–18°
*b* = 8.2407 (16) Å	µ = 0.20 mm^−^^1^
*c* = 33.083 (7) Å	*T* = 293 K
β = 93.17 (3)°	Needle, colourless
*V* = 2070.6 (7) Å^3^	0.46 × 0.08 × 0.08 mm
*Z* = 2	

### Data collection

**Table:**

Bruker SMART 1K CCD diffractometer	8425 independent reflections
Radiation source: fine-focus sealed tube	2925 reflections with *I* > 2σ(*I*)
graphite	*R*_int_ = 0.106
ω scans	θ_max_ = 28.3°, θ_min_ = 0.6°
Absorption correction: multi-scan (*SADABS*; Bruker, 1998)	*h* = −10→9
*T*_min_ = 0.914, *T*_max_ = 0.984	*k* = −8→10
14589 measured reflections	*l* = −44→41

### Refinement

**Table:**

Refinement on *F*^2^	H atoms treated by a mixture of independent and constrained refinement
Least-squares matrix: full	*w* = 1/[σ^2^(*F*_o_^2^) + (0.0465*P*)^2^ + 0.3561*P*] where *P* = (*F*_o_^2^ + 2*F*_c_^2^)/3
*R*[*F*^2^ > 2σ(*F*^2^)] = 0.066	(Δ/σ)_max_ = 0.001
*wR*(*F*^2^) = 0.159	Δρ_max_ = 0.29 e Å^−^^3^
*S* = 0.93	Δρ_min_ = −0.28 e Å^−^^3^
8425 reflections	Absolute structure: Flack (1983), 2970 Friedel pairs
410 parameters	Flack parameter: 0.02 (12)
1 restraint	

### Special details

**Table:**

Experimental. The intensity data was collected on a Bruker *SMART* 1 K CCD diffractometer using an exposure time of 10 s/frame. A total of 1315 frames were collected with a frame width of 0.3° covering up to θ = 28.3° with 99.8% completeness accomplished.
Geometry. All e.s.d.'s (except the e.s.d. in the dihedral angle between two l.s. planes) are estimated using the full covariance matrix. The cell e.s.d.'s are taken into account individually in the estimation of e.s.d.'s in distances, angles and torsion angles; correlations between e.s.d.'s in cell parameters are only used when they are defined by crystal symmetry. An approximate (isotropic) treatment of cell e.s.d.'s is used for estimating e.s.d.'s involving l.s. planes.

### Fractional atomic coordinates and isotropic or equivalent isotropic displacement parameters (Å^2^)

**Table:**

	*x*	*y*	*z*	*U*_iso_*/*U*_eq_	
P	0.4405 (2)	0.9520 (2)	0.64139 (5)	0.0564 (5)	
S1	0.6976 (2)	0.9298 (2)	0.63620 (6)	0.0765 (6)	
S2	0.3618 (2)	1.1632 (2)	0.66205 (5)	0.0787 (6)	
N	0.7566 (8)	0.3414 (8)	0.64246 (16)	0.0629 (16)	
O1	0.3661 (5)	0.8058 (4)	0.66822 (10)	0.0582 (12)	
O2	0.0389 (9)	0.7700 (7)	0.48803 (16)	0.117 (2)	
C1	0.3206 (8)	0.8992 (7)	0.59454 (18)	0.0553 (17)	
C2	0.4030 (9)	0.8534 (9)	0.5604 (2)	0.089 (3)	
H2	0.5253	0.8519	0.5605	0.107*	
C3	0.3002 (12)	0.8082 (11)	0.5251 (2)	0.108 (3)	
H3	0.3555	0.7742	0.5021	0.13*	
C4	0.1217 (11)	0.8144 (9)	0.5245 (2)	0.078 (2)	
C5	0.0390 (9)	0.8652 (7)	0.5579 (2)	0.0653 (19)	
H5	−0.0831	0.8733	0.5571	0.078*	
C6	0.1382 (9)	0.9046 (7)	0.59280 (19)	0.0632 (18)	
H6	0.081	0.9354	0.6157	0.076*	
C7	−0.1478 (13)	0.7738 (12)	0.4851 (2)	0.130 (3)	
H7A	−0.1928	0.6977	0.5039	0.195*	
H7B	−0.1893	0.7453	0.4581	0.195*	
H7C	−0.1878	0.881	0.4913	0.195*	
C8	0.3646 (8)	0.8128 (7)	0.71259 (16)	0.0540 (16)	
H8	0.4108	0.9181	0.722	0.065*	
C9	0.1781 (8)	0.7960 (9)	0.72416 (17)	0.073 (2)	
H9A	0.1281	0.6959	0.7132	0.087*	
H9B	0.1082	0.8859	0.7132	0.087*	
C10	0.1755 (8)	0.7948 (8)	0.77036 (17)	0.0672 (19)	
H10A	0.0545	0.785	0.7778	0.081*	
H10B	0.2202	0.8979	0.7806	0.081*	
C11	0.2848 (7)	0.6567 (8)	0.79104 (15)	0.0478 (15)	
C12	0.4698 (8)	0.6673 (7)	0.77557 (16)	0.0491 (15)	
C13	0.4797 (7)	0.6801 (8)	0.73037 (16)	0.0618 (17)	
H13A	0.6007	0.7006	0.724	0.074*	
H13B	0.4442	0.5775	0.7181	0.074*	
C14	0.2057 (9)	0.4918 (8)	0.78016 (18)	0.085 (2)	
H14A	0.2334	0.4635	0.7531	0.127*	
H14B	0.0802	0.4962	0.7818	0.127*	
H14C	0.2537	0.4115	0.7987	0.127*	
C15	0.2930 (6)	0.6876 (7)	0.83762 (16)	0.0490 (16)	
H15	0.3168	0.8036	0.8415	0.059*	
C16	0.4436 (7)	0.5964 (7)	0.86033 (15)	0.0474 (15)	
H16	0.4254	0.4797	0.8562	0.057*	
C17	0.6186 (6)	0.6417 (8)	0.84456 (16)	0.0597 (17)	
H17A	0.6575	0.7433	0.8568	0.072*	
H17B	0.7043	0.5591	0.8526	0.072*	
C18	0.6129 (8)	0.6594 (8)	0.79945 (17)	0.0557 (16)	
H18	0.7202	0.6653	0.7873	0.067*	
C19	0.1159 (6)	0.6524 (8)	0.85715 (15)	0.0594 (16)	
H19A	0.026	0.7225	0.8447	0.071*	
H19B	0.0819	0.5412	0.8511	0.071*	
C20	0.1201 (7)	0.6770 (8)	0.90307 (15)	0.0554 (16)	
H20A	0.0083	0.643	0.913	0.067*	
H20B	0.135	0.7915	0.9091	0.067*	
C21	0.2691 (7)	0.5811 (7)	0.92521 (16)	0.0449 (15)	
C22	0.4385 (6)	0.6324 (7)	0.90520 (14)	0.0423 (14)	
H22	0.4434	0.7509	0.9075	0.051*	
C23	0.2355 (8)	0.3976 (7)	0.92165 (15)	0.0570 (17)	
H23A	0.2346	0.366	0.8937	0.085*	
H23B	0.1239	0.3722	0.9323	0.085*	
H23C	0.3271	0.3401	0.9368	0.085*	
C24	0.3149 (6)	0.6274 (6)	0.96991 (15)	0.0433 (14)	
H24	0.3076	0.7459	0.9716	0.052*	
C25	0.5127 (7)	0.5825 (7)	0.97587 (16)	0.0527 (16)	
H25A	0.5753	0.6655	0.9917	0.063*	
H25B	0.5259	0.48	0.9901	0.063*	
C26	0.5878 (7)	0.5689 (8)	0.93369 (17)	0.0583 (17)	
H26A	0.6928	0.6348	0.932	0.07*	
H26B	0.6161	0.4572	0.9274	0.07*	
C27	0.2062 (7)	0.5588 (7)	1.00409 (16)	0.0478 (15)	
H27	0.2239	0.4411	1.0047	0.057*	
C28	0.0093 (8)	0.5893 (7)	0.99762 (17)	0.0617 (18)	
H28A	−0.049	0.5552	1.0212	0.093*	
H28B	−0.0359	0.5289	0.9745	0.093*	
H28C	−0.0112	0.7029	0.9931	0.093*	
C29	0.2764 (8)	0.6252 (8)	1.04439 (15)	0.0656 (19)	
H29A	0.2584	0.7418	1.044	0.079*	
H29B	0.4025	0.6069	1.0464	0.079*	
C30	0.2014 (8)	0.5599 (7)	1.08297 (16)	0.0611 (18)	
H30A	0.0777	0.5885	1.0832	0.073*	
H30B	0.2099	0.4425	1.0831	0.073*	
C31	0.2976 (11)	0.6267 (10)	1.12087 (19)	0.1104 (14)	
H31A	0.4189	0.5903	1.1211	0.132*	
H31B	0.2988	0.7441	1.1187	0.132*	
C32	0.2273 (11)	0.5835 (10)	1.1606 (2)	0.1104 (14)	
H32	0.2151	0.4651	1.1612	0.132*	
C33	0.0500 (10)	0.6538 (10)	1.16635 (19)	0.1104 (14)	
H33A	0.0569	0.7701	1.1655	0.166*	
H33B	0.0104	0.6204	1.1921	0.166*	
H33C	−0.0314	0.6163	1.1452	0.166*	
C34	0.3558 (10)	0.6306 (10)	1.19511 (18)	0.1104 (14)	
H34A	0.3734	0.7459	1.1949	0.166*	
H34B	0.4662	0.5768	1.192	0.166*	
H34C	0.3093	0.599	1.2203	0.166*	
C35	0.9219 (8)	0.3208 (8)	0.62051 (19)	0.0705 (19)	
H35A	0.8913	0.2858	0.593	0.085*	
H35B	0.9926	0.236	0.6337	0.085*	
C36	1.0308 (9)	0.4735 (10)	0.6191 (2)	0.115 (3)	
H36A	0.9634	0.5571	0.6052	0.172*	
H36B	1.1353	0.4522	0.605	0.172*	
H36C	1.0631	0.5084	0.6462	0.172*	
C37	0.7919 (10)	0.3745 (9)	0.6876 (2)	0.091 (2)	
H37A	0.8507	0.4785	0.6907	0.109*	
H37B	0.6799	0.3828	0.7001	0.109*	
C38	0.9001 (11)	0.2499 (10)	0.7094 (2)	0.109 (3)	
H38A	0.8464	0.1454	0.7053	0.164*	
H38B	0.9083	0.2748	0.7378	0.164*	
H38C	1.0159	0.2488	0.6992	0.164*	
C39	0.6301 (10)	0.4630 (10)	0.6243 (2)	0.099 (2)	
H39A	0.6847	0.5693	0.6252	0.119*	
H39B	0.527	0.4674	0.6402	0.119*	
C40	0.5737 (11)	0.4232 (12)	0.5813 (2)	0.130 (3)	
H40A	0.6695	0.4434	0.5642	0.195*	
H40B	0.475	0.4898	0.5727	0.195*	
H40C	0.5406	0.3109	0.5794	0.195*	
H1	0.705 (9)	0.235 (8)	0.6428 (19)	0.11 (3)*	

### Atomic displacement parameters (Å^2^)

**Table:**

	*U*^11^	*U*^22^	*U*^33^	*U*^12^	*U*^13^	*U*^23^
P	0.0556 (11)	0.0558 (12)	0.0586 (11)	−0.0041 (10)	0.0114 (9)	0.0045 (10)
S1	0.0517 (11)	0.0771 (14)	0.1017 (15)	−0.0014 (10)	0.0143 (10)	−0.0089 (11)
S2	0.0778 (13)	0.0637 (12)	0.0961 (14)	0.0056 (11)	0.0175 (11)	−0.0043 (11)
N	0.068 (4)	0.053 (4)	0.068 (4)	0.000 (4)	0.008 (3)	−0.003 (3)
O1	0.073 (3)	0.060 (3)	0.042 (3)	−0.016 (2)	0.007 (2)	0.007 (2)
O2	0.106 (5)	0.182 (6)	0.060 (4)	0.025 (4)	−0.010 (3)	−0.019 (3)
C1	0.053 (4)	0.063 (4)	0.050 (4)	0.002 (3)	0.006 (3)	0.017 (3)
C2	0.069 (5)	0.153 (8)	0.046 (5)	0.021 (5)	0.012 (4)	0.010 (4)
C3	0.098 (7)	0.177 (9)	0.051 (6)	0.027 (6)	0.016 (5)	−0.028 (5)
C4	0.070 (6)	0.107 (6)	0.058 (6)	0.012 (5)	−0.006 (5)	0.002 (4)
C5	0.065 (5)	0.078 (5)	0.054 (4)	0.009 (4)	0.007 (4)	0.003 (4)
C6	0.063 (5)	0.072 (5)	0.055 (5)	−0.007 (4)	0.006 (4)	−0.001 (4)
C7	0.120 (9)	0.177 (10)	0.087 (6)	−0.010 (7)	−0.037 (6)	−0.024 (6)
C8	0.058 (4)	0.064 (4)	0.041 (4)	0.001 (4)	0.009 (3)	−0.001 (3)
C9	0.056 (5)	0.111 (6)	0.052 (5)	0.012 (4)	0.008 (4)	0.013 (4)
C10	0.046 (4)	0.106 (6)	0.051 (4)	0.011 (4)	0.004 (3)	0.009 (4)
C11	0.044 (4)	0.066 (4)	0.034 (3)	−0.014 (4)	0.004 (3)	0.008 (3)
C12	0.044 (4)	0.060 (4)	0.044 (4)	0.005 (3)	0.007 (3)	0.002 (3)
C13	0.051 (4)	0.077 (5)	0.058 (4)	0.002 (4)	0.010 (3)	0.005 (4)
C14	0.094 (5)	0.105 (6)	0.055 (4)	−0.042 (5)	0.003 (4)	0.000 (4)
C15	0.027 (3)	0.067 (4)	0.053 (4)	0.001 (3)	0.005 (3)	0.006 (3)
C16	0.036 (4)	0.059 (4)	0.047 (4)	−0.001 (3)	−0.003 (3)	−0.001 (3)
C17	0.033 (4)	0.087 (5)	0.060 (4)	0.002 (4)	0.008 (3)	0.014 (4)
C18	0.040 (4)	0.072 (4)	0.056 (4)	0.004 (4)	0.009 (3)	0.006 (4)
C19	0.033 (4)	0.089 (5)	0.056 (4)	0.007 (4)	−0.003 (3)	0.010 (4)
C20	0.034 (4)	0.087 (5)	0.046 (4)	0.003 (4)	0.008 (3)	0.012 (4)
C21	0.039 (4)	0.051 (4)	0.045 (4)	0.001 (3)	−0.001 (3)	0.000 (3)
C22	0.032 (3)	0.053 (4)	0.041 (4)	0.000 (3)	−0.006 (3)	0.001 (3)
C23	0.068 (4)	0.059 (5)	0.044 (4)	−0.012 (3)	0.005 (3)	−0.003 (3)
C24	0.046 (4)	0.037 (4)	0.047 (4)	0.002 (3)	0.000 (3)	0.000 (3)
C25	0.043 (4)	0.064 (4)	0.049 (4)	−0.005 (3)	−0.009 (3)	0.000 (3)
C26	0.035 (4)	0.082 (4)	0.058 (4)	0.008 (3)	0.005 (3)	0.010 (3)
C27	0.048 (4)	0.044 (4)	0.051 (4)	−0.005 (3)	0.000 (3)	−0.001 (3)
C28	0.059 (5)	0.065 (5)	0.062 (4)	0.003 (3)	0.019 (3)	0.004 (3)
C29	0.079 (5)	0.074 (5)	0.044 (4)	−0.019 (4)	0.007 (3)	−0.003 (4)
C30	0.084 (5)	0.063 (4)	0.036 (4)	−0.006 (4)	0.006 (3)	0.000 (3)
C31	0.152 (4)	0.123 (4)	0.056 (2)	0.010 (3)	0.008 (3)	−0.006 (3)
C32	0.152 (4)	0.123 (4)	0.056 (2)	0.010 (3)	0.008 (3)	−0.006 (3)
C33	0.152 (4)	0.123 (4)	0.056 (2)	0.010 (3)	0.008 (3)	−0.006 (3)
C34	0.152 (4)	0.123 (4)	0.056 (2)	0.010 (3)	0.008 (3)	−0.006 (3)
C35	0.068 (5)	0.073 (5)	0.071 (5)	0.002 (4)	0.010 (4)	−0.005 (4)
C36	0.091 (6)	0.086 (6)	0.171 (8)	−0.005 (5)	0.044 (5)	0.026 (6)
C37	0.103 (6)	0.091 (6)	0.079 (6)	−0.020 (5)	0.016 (5)	−0.034 (4)
C38	0.111 (7)	0.132 (8)	0.084 (6)	−0.014 (6)	−0.008 (5)	0.007 (5)
C39	0.088 (6)	0.074 (5)	0.135 (7)	0.007 (5)	0.004 (5)	0.017 (6)
C40	0.116 (7)	0.167 (9)	0.105 (7)	0.010 (7)	−0.011 (6)	0.054 (7)

### Geometric parameters (Å, °)

**Table:**

P—O1	1.617 (4)	C21—C23	1.537 (7)
P—C1	1.807 (6)	C21—C22	1.540 (7)
P—S2	1.975 (2)	C21—C24	1.548 (7)
P—S1	1.981 (2)	C22—C26	1.528 (7)
N—C39	1.493 (8)	C22—H22	0.98
N—C35	1.496 (7)	C23—H23A	0.96
N—C37	1.526 (8)	C23—H23B	0.96
N—H1	0.96 (7)	C23—H23C	0.96
O1—C8	1.470 (6)	C24—C27	1.545 (7)
O2—C4	1.379 (8)	C24—C25	1.551 (7)
O2—C7	1.419 (9)	C24—H24	0.98
C1—C2	1.374 (8)	C25—C26	1.541 (7)
C1—C6	1.387 (7)	C25—H25A	0.97
C2—C3	1.421 (9)	C25—H25B	0.97
C2—H2	0.93	C26—H26A	0.97
C3—C4	1.357 (9)	C26—H26B	0.97
C3—H3	0.93	C27—C29	1.511 (7)
C4—C5	1.367 (8)	C27—C28	1.522 (7)
C5—C6	1.383 (8)	C27—H27	0.98
C5—H5	0.93	C28—H28A	0.96
C6—H6	0.93	C28—H28B	0.96
C7—H7A	0.96	C28—H28C	0.96
C7—H7B	0.96	C29—C30	1.525 (7)
C7—H7C	0.96	C29—H29A	0.97
C8—C9	1.496 (7)	C29—H29B	0.97
C8—C13	1.501 (7)	C30—C31	1.520 (8)
C8—H8	0.98	C30—H30A	0.97
C9—C10	1.530 (7)	C30—H30B	0.97
C9—H9A	0.97	C31—C32	1.488 (9)
C9—H9B	0.97	C31—H31A	0.97
C10—C11	1.546 (8)	C31—H31B	0.97
C10—H10A	0.97	C32—C33	1.490 (9)
C10—H10B	0.97	C32—C34	1.512 (9)
C11—C14	1.521 (8)	C32—H32	0.98
C11—C12	1.527 (7)	C33—H33A	0.96
C11—C15	1.559 (7)	C33—H33B	0.96
C12—C18	1.311 (7)	C33—H33C	0.96
C12—C13	1.505 (7)	C34—H34A	0.96
C13—H13A	0.97	C34—H34B	0.96
C13—H13B	0.97	C34—H34C	0.96
C14—H14A	0.96	C35—C36	1.508 (9)
C14—H14B	0.96	C35—H35A	0.97
C14—H14C	0.96	C35—H35B	0.97
C15—C16	1.532 (7)	C36—H36A	0.96
C15—C19	1.553 (6)	C36—H36B	0.96
C15—H15	0.98	C36—H36C	0.96
C16—C17	1.503 (6)	C37—C38	1.479 (9)
C16—C22	1.516 (6)	C37—H37A	0.97
C16—H16	0.98	C37—H37B	0.97
C17—C18	1.498 (7)	C38—H38A	0.96
C17—H17A	0.97	C38—H38B	0.96
C17—H17B	0.97	C38—H38C	0.96
C18—H18	0.93	C39—C40	1.498 (9)
C19—C20	1.531 (6)	C39—H39A	0.97
C19—H19A	0.97	C39—H39B	0.97
C19—H19B	0.97	C40—H40A	0.96
C20—C21	1.534 (7)	C40—H40B	0.96
C20—H20A	0.97	C40—H40C	0.96
C20—H20B	0.97		
			
O1—P—C1	96.7 (2)	C22—C21—C24	101.2 (4)
O1—P—S2	110.16 (16)	C16—C22—C26	118.6 (4)
C1—P—S2	111.1 (2)	C16—C22—C21	115.8 (4)
O1—P—S1	110.86 (17)	C26—C22—C21	104.6 (4)
C1—P—S1	110.9 (2)	C16—C22—H22	105.6
S2—P—S1	115.52 (11)	C26—C22—H22	105.6
C39—N—C35	114.9 (5)	C21—C22—H22	105.6
C39—N—C37	110.4 (6)	C21—C23—H23A	109.5
C35—N—C37	112.8 (5)	C21—C23—H23B	109.5
C39—N—H1	111 (4)	H23A—C23—H23B	109.5
C35—N—H1	105 (4)	C21—C23—H23C	109.5
C37—N—H1	102 (4)	H23A—C23—H23C	109.5
C8—O1—P	122.8 (3)	H23B—C23—H23C	109.5
C4—O2—C7	117.4 (6)	C27—C24—C21	120.5 (4)
C2—C1—C6	118.4 (6)	C27—C24—C25	112.0 (4)
C2—C1—P	122.6 (5)	C21—C24—C25	103.2 (4)
C6—C1—P	119.0 (5)	C27—C24—H24	106.8
C1—C2—C3	119.6 (7)	C21—C24—H24	106.8
C1—C2—H2	120.2	C25—C24—H24	106.8
C3—C2—H2	120.2	C26—C25—C24	107.9 (4)
C4—C3—C2	120.4 (7)	C26—C25—H25A	110.1
C4—C3—H3	119.8	C24—C25—H25A	110.1
C2—C3—H3	119.8	C26—C25—H25B	110.1
C3—C4—C5	120.3 (7)	C24—C25—H25B	110.1
C3—C4—O2	114.3 (7)	H25A—C25—H25B	108.4
C5—C4—O2	125.4 (7)	C22—C26—C25	103.5 (4)
C4—C5—C6	119.5 (7)	C22—C26—H26A	111.1
C4—C5—H5	120.2	C25—C26—H26A	111.1
C6—C5—H5	120.2	C22—C26—H26B	111.1
C5—C6—C1	121.7 (6)	C25—C26—H26B	111.1
C5—C6—H6	119.2	H26A—C26—H26B	109
C1—C6—H6	119.2	C29—C27—C28	111.2 (5)
O2—C7—H7A	109.5	C29—C27—C24	109.7 (5)
O2—C7—H7B	109.5	C28—C27—C24	113.5 (5)
H7A—C7—H7B	109.5	C29—C27—H27	107.4
O2—C7—H7C	109.5	C28—C27—H27	107.4
H7A—C7—H7C	109.5	C24—C27—H27	107.4
H7B—C7—H7C	109.5	C27—C28—H28A	109.5
O1—C8—C9	108.2 (5)	C27—C28—H28B	109.5
O1—C8—C13	109.0 (4)	H28A—C28—H28B	109.5
C9—C8—C13	111.9 (5)	C27—C28—H28C	109.5
O1—C8—H8	109.2	H28A—C28—H28C	109.5
C9—C8—H8	109.2	H28B—C28—H28C	109.5
C13—C8—H8	109.2	C27—C29—C30	118.7 (5)
C8—C9—C10	108.7 (5)	C27—C29—H29A	107.6
C8—C9—H9A	109.9	C30—C29—H29A	107.6
C10—C9—H9A	109.9	C27—C29—H29B	107.6
C8—C9—H9B	109.9	C30—C29—H29B	107.6
C10—C9—H9B	109.9	H29A—C29—H29B	107.1
H9A—C9—H9B	108.3	C31—C30—C29	112.2 (5)
C9—C10—C11	114.2 (5)	C31—C30—H30A	109.2
C9—C10—H10A	108.7	C29—C30—H30A	109.2
C11—C10—H10A	108.7	C31—C30—H30B	109.2
C9—C10—H10B	108.7	C29—C30—H30B	109.2
C11—C10—H10B	108.7	H30A—C30—H30B	107.9
H10A—C10—H10B	107.6	C32—C31—C30	117.5 (7)
C14—C11—C12	109.3 (5)	C32—C31—H31A	107.9
C14—C11—C10	110.9 (5)	C30—C31—H31A	107.9
C12—C11—C10	107.0 (5)	C32—C31—H31B	107.9
C14—C11—C15	111.9 (4)	C30—C31—H31B	107.9
C12—C11—C15	109.6 (4)	H31A—C31—H31B	107.2
C10—C11—C15	108.0 (5)	C31—C32—C33	113.1 (7)
C18—C12—C13	121.1 (5)	C31—C32—C34	110.9 (7)
C18—C12—C11	123.1 (5)	C33—C32—C34	110.8 (6)
C13—C12—C11	115.8 (5)	C31—C32—H32	107.3
C8—C13—C12	112.3 (5)	C33—C32—H32	107.3
C8—C13—H13A	109.1	C34—C32—H32	107.3
C12—C13—H13A	109.1	C32—C33—H33A	109.5
C8—C13—H13B	109.1	C32—C33—H33B	109.5
C12—C13—H13B	109.1	H33A—C33—H33B	109.5
H13A—C13—H13B	107.9	C32—C33—H33C	109.5
C11—C14—H14A	109.5	H33A—C33—H33C	109.5
C11—C14—H14B	109.5	H33B—C33—H33C	109.5
H14A—C14—H14B	109.5	C32—C34—H34A	109.5
C11—C14—H14C	109.5	C32—C34—H34B	109.5
H14A—C14—H14C	109.5	H34A—C34—H34B	109.5
H14B—C14—H14C	109.5	C32—C34—H34C	109.5
C16—C15—C19	110.3 (4)	H34A—C34—H34C	109.5
C16—C15—C11	113.1 (4)	H34B—C34—H34C	109.5
C19—C15—C11	113.1 (4)	N—C35—C36	113.5 (6)
C16—C15—H15	106.6	N—C35—H35A	108.9
C19—C15—H15	106.6	C36—C35—H35A	108.9
C11—C15—H15	106.6	N—C35—H35B	108.9
C17—C16—C22	111.3 (4)	C36—C35—H35B	108.9
C17—C16—C15	111.1 (4)	H35A—C35—H35B	107.7
C22—C16—C15	109.0 (4)	C35—C36—H36A	109.5
C17—C16—H16	108.5	C35—C36—H36B	109.5
C22—C16—H16	108.5	H36A—C36—H36B	109.5
C15—C16—H16	108.5	C35—C36—H36C	109.5
C18—C17—C16	113.1 (5)	H36A—C36—H36C	109.5
C18—C17—H17A	108.9	H36B—C36—H36C	109.5
C16—C17—H17A	108.9	C38—C37—N	114.6 (6)
C18—C17—H17B	108.9	C38—C37—H37A	108.6
C16—C17—H17B	108.9	N—C37—H37A	108.6
H17A—C17—H17B	107.8	C38—C37—H37B	108.6
C12—C18—C17	125.6 (5)	N—C37—H37B	108.6
C12—C18—H18	117.2	H37A—C37—H37B	107.6
C17—C18—H18	117.2	C37—C38—H38A	109.5
C20—C19—C15	114.7 (4)	C37—C38—H38B	109.5
C20—C19—H19A	108.6	H38A—C38—H38B	109.5
C15—C19—H19A	108.6	C37—C38—H38C	109.5
C20—C19—H19B	108.6	H38A—C38—H38C	109.5
C15—C19—H19B	108.6	H38B—C38—H38C	109.5
H19A—C19—H19B	107.6	N—C39—C40	112.3 (7)
C19—C20—C21	112.3 (5)	N—C39—H39A	109.1
C19—C20—H20A	109.1	C40—C39—H39A	109.1
C21—C20—H20A	109.1	N—C39—H39B	109.1
C19—C20—H20B	109.1	C40—C39—H39B	109.1
C21—C20—H20B	109.1	H39A—C39—H39B	107.9
H20A—C20—H20B	107.9	C39—C40—H40A	109.5
C20—C21—C23	110.8 (5)	C39—C40—H40B	109.5
C20—C21—C22	105.5 (4)	H40A—C40—H40B	109.5
C23—C21—C22	112.1 (5)	C39—C40—H40C	109.5
C20—C21—C24	116.8 (5)	H40A—C40—H40C	109.5
C23—C21—C24	110.1 (4)	H40B—C40—H40C	109.5
			
C1—P—O1—C8	−157.5 (4)	C22—C16—C17—C18	−161.6 (5)
S2—P—O1—C8	−42.0 (4)	C15—C16—C17—C18	−40.0 (7)
S1—P—O1—C8	87.1 (4)	C13—C12—C18—C17	−178.1 (6)
O1—P—C1—C2	−116.5 (6)	C11—C12—C18—C17	−0.9 (11)
S2—P—C1—C2	128.9 (5)	C16—C17—C18—C12	12.8 (9)
S1—P—C1—C2	−1.1 (6)	C16—C15—C19—C20	50.7 (7)
O1—P—C1—C6	63.4 (5)	C11—C15—C19—C20	178.5 (5)
S2—P—C1—C6	−51.3 (5)	C15—C19—C20—C21	−53.6 (7)
S1—P—C1—C6	178.7 (4)	C19—C20—C21—C23	−67.2 (6)
C6—C1—C2—C3	−2.0 (10)	C19—C20—C21—C22	54.3 (6)
P—C1—C2—C3	177.9 (6)	C19—C20—C21—C24	165.7 (5)
C1—C2—C3—C4	1.8 (13)	C17—C16—C22—C26	−50.9 (7)
C2—C3—C4—C5	0.4 (13)	C15—C16—C22—C26	−173.7 (5)
C2—C3—C4—O2	178.9 (7)	C17—C16—C22—C21	−176.4 (5)
C7—O2—C4—C3	−179.9 (8)	C15—C16—C22—C21	60.7 (6)
C7—O2—C4—C5	−1.5 (12)	C20—C21—C22—C16	−60.7 (6)
C3—C4—C5—C6	−2.4 (11)	C23—C21—C22—C16	59.9 (6)
O2—C4—C5—C6	179.3 (6)	C24—C21—C22—C16	177.2 (5)
C4—C5—C6—C1	2.2 (9)	C20—C21—C22—C26	166.9 (5)
C2—C1—C6—C5	0.0 (9)	C23—C21—C22—C26	−72.5 (5)
P—C1—C6—C5	−179.9 (5)	C24—C21—C22—C26	44.8 (5)
P—O1—C8—C9	120.6 (5)	C20—C21—C24—C27	82.4 (6)
P—O1—C8—C13	−117.5 (5)	C23—C21—C24—C27	−45.1 (7)
O1—C8—C9—C10	177.3 (5)	C22—C21—C24—C27	−163.8 (5)
C13—C8—C9—C10	57.2 (7)	C20—C21—C24—C25	−151.9 (5)
C8—C9—C10—C11	−59.0 (7)	C23—C21—C24—C25	80.7 (5)
C9—C10—C11—C14	−65.7 (6)	C22—C21—C24—C25	−38.0 (5)
C9—C10—C11—C12	53.5 (7)	C27—C24—C25—C26	149.7 (5)
C9—C10—C11—C15	171.4 (5)	C21—C24—C25—C26	18.6 (6)
C14—C11—C12—C18	−106.3 (7)	C16—C22—C26—C25	−163.8 (5)
C10—C11—C12—C18	133.5 (6)	C21—C22—C26—C25	−33.0 (6)
C15—C11—C12—C18	16.7 (9)	C24—C25—C26—C22	8.6 (6)
C14—C11—C12—C13	71.1 (6)	C21—C24—C27—C29	−179.0 (5)
C10—C11—C12—C13	−49.1 (7)	C25—C24—C27—C29	59.4 (6)
C15—C11—C12—C13	−166.0 (5)	C21—C24—C27—C28	−54.0 (7)
O1—C8—C13—C12	−173.7 (5)	C25—C24—C27—C28	−175.6 (5)
C9—C8—C13—C12	−54.1 (7)	C28—C27—C29—C30	60.0 (7)
C18—C12—C13—C8	−131.3 (6)	C24—C27—C29—C30	−173.6 (5)
C11—C12—C13—C8	51.3 (7)	C27—C29—C30—C31	174.5 (6)
C14—C11—C15—C16	76.9 (6)	C29—C30—C31—C32	174.9 (6)
C12—C11—C15—C16	−44.6 (7)	C30—C31—C32—C33	−66.0 (9)
C10—C11—C15—C16	−160.8 (5)	C30—C31—C32—C34	168.9 (6)
C14—C11—C15—C19	−49.4 (7)	C39—N—C35—C36	61.5 (8)
C12—C11—C15—C19	−170.9 (5)	C37—N—C35—C36	−66.2 (8)
C10—C11—C15—C19	72.9 (6)	C39—N—C37—C38	173.5 (7)
C19—C15—C16—C17	−174.3 (5)	C35—N—C37—C38	−56.5 (8)
C11—C15—C16—C17	57.9 (6)	C35—N—C39—C40	58.3 (8)
C19—C15—C16—C22	−51.4 (6)	C37—N—C39—C40	−172.8 (6)
C11—C15—C16—C22	−179.2 (4)		

### Hydrogen-bond geometry (Å, °)

**Table:**

*D*—H···*A*	*D*—H	H···*A*	*D*···*A*	*D*—H···*A*
N—H1···S1^i^	0.96 (7)	2.53 (7)	3.426 (7)	156 (5)
N—H1···S2^i^	0.96 (7)	2.78 (7)	3.437 (6)	126 (5)

Symmetry codes: (i) *x*, *y*−1, *z*.
